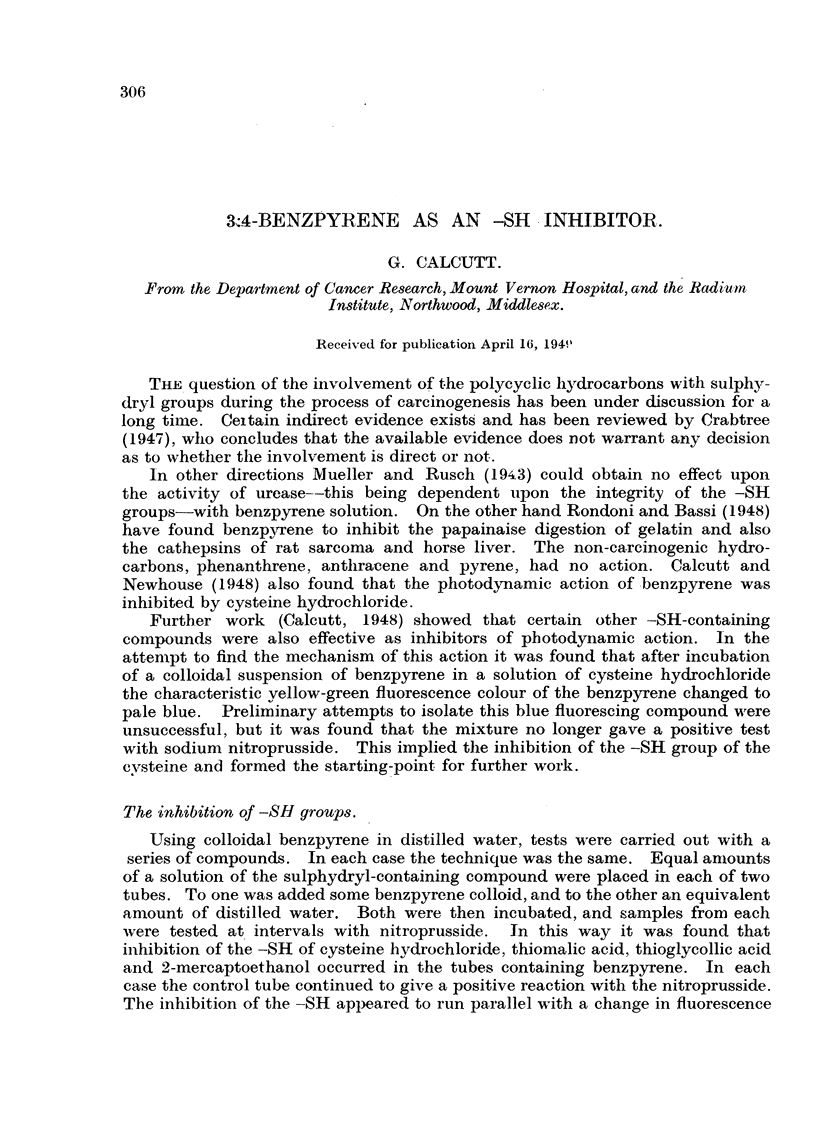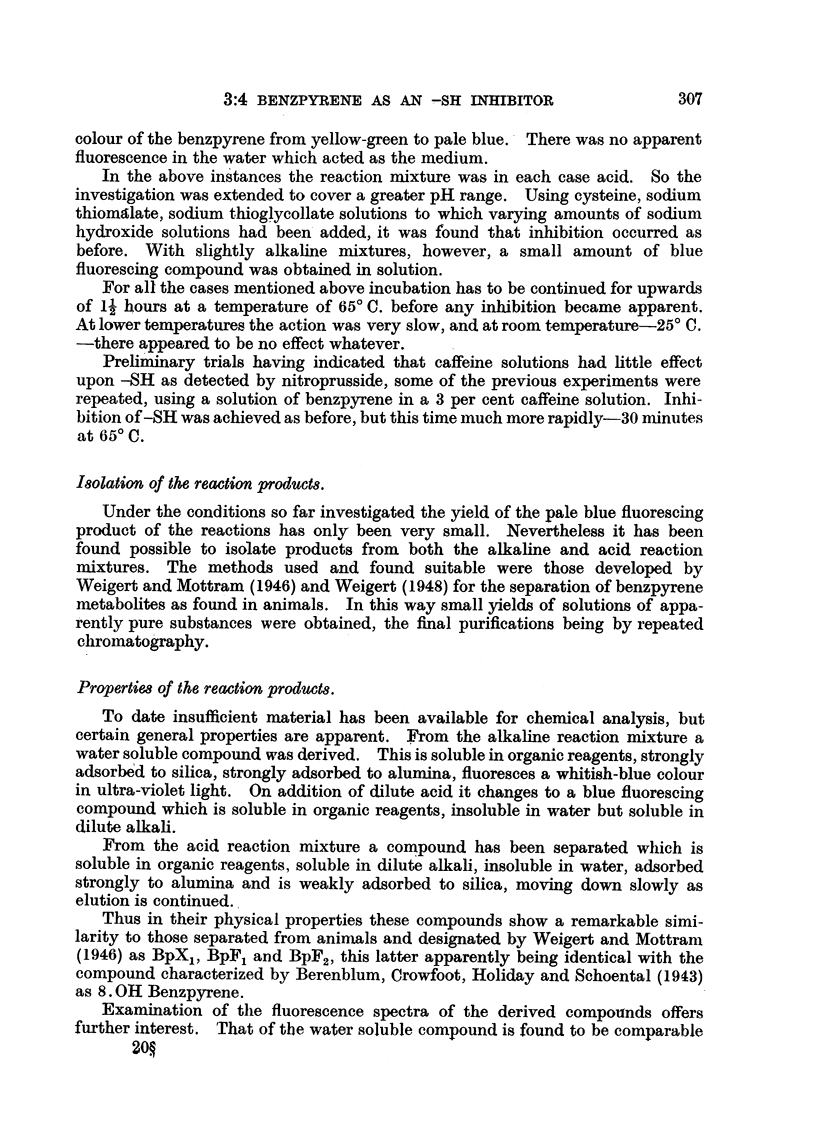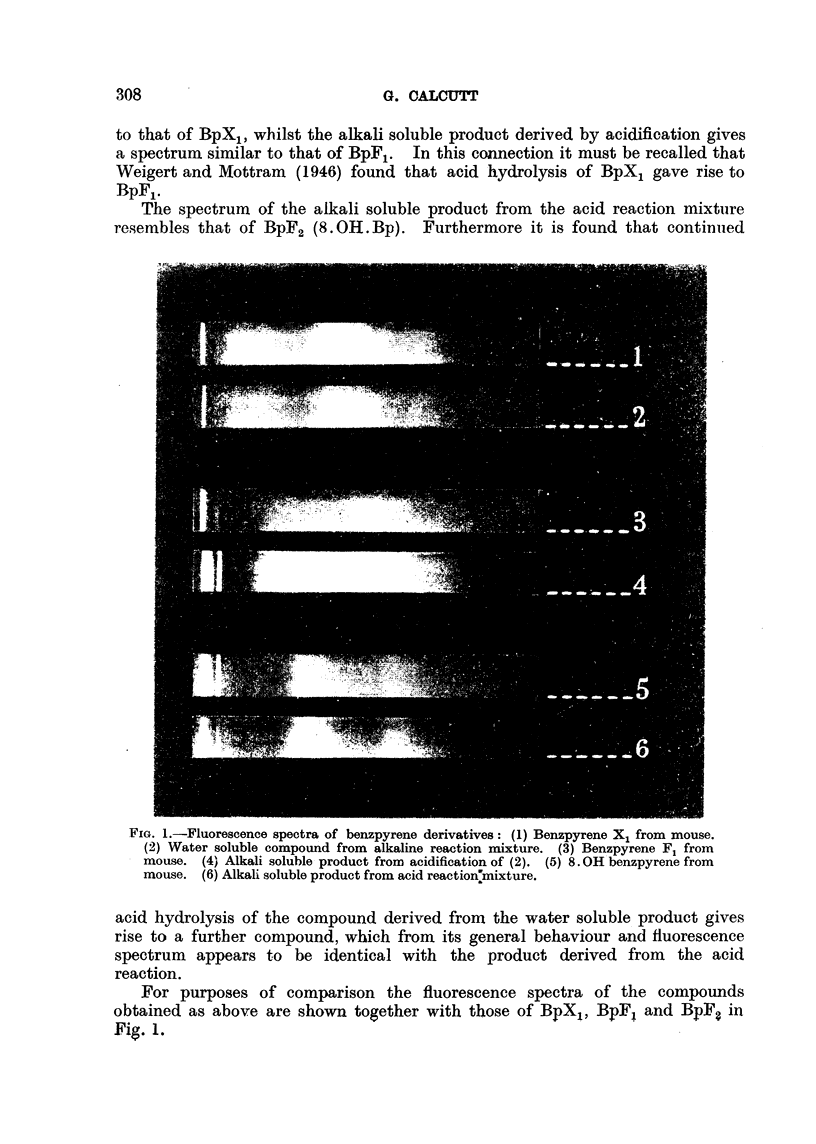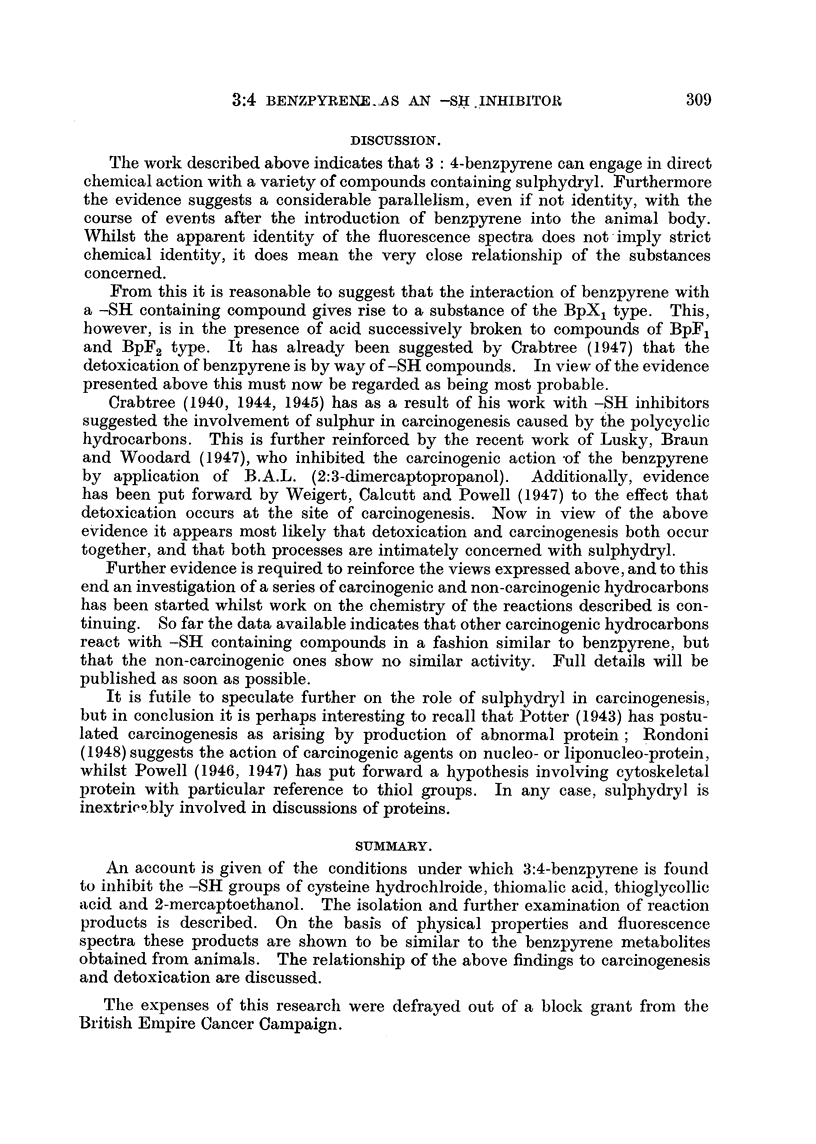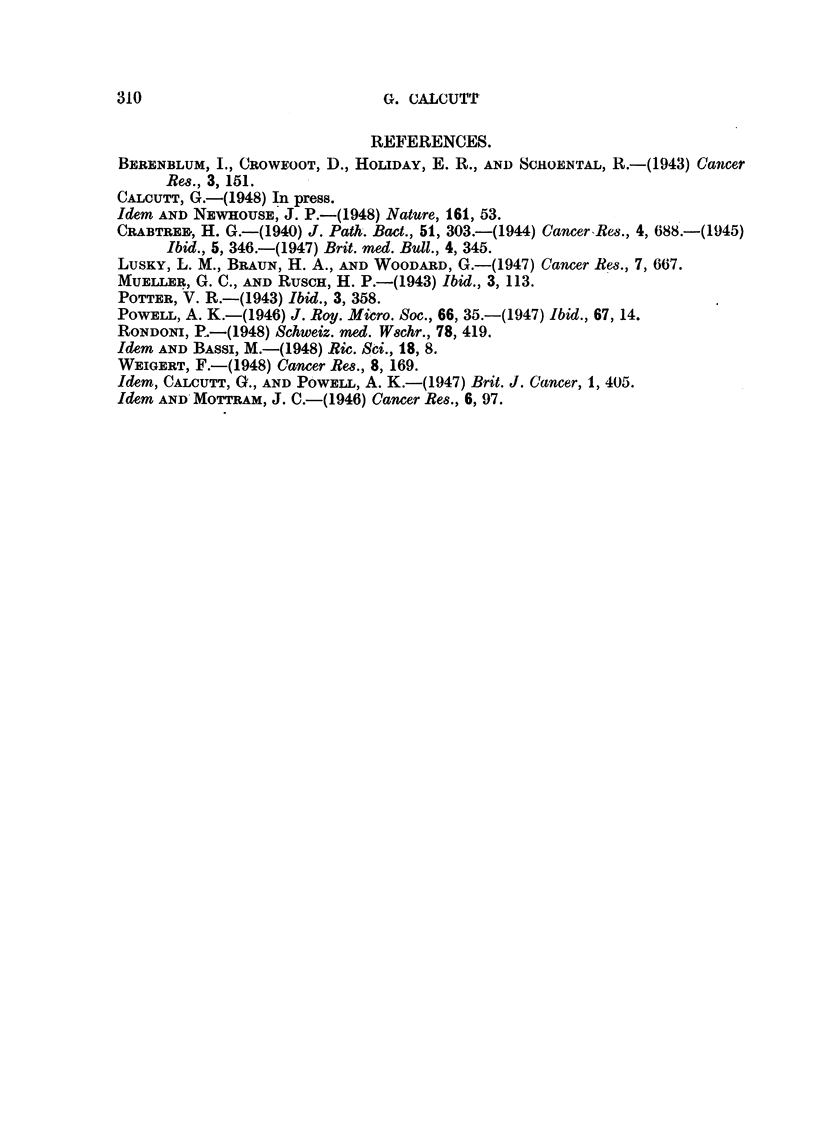# 3:4-Benzpyrene as an -SH Inhibitor

**DOI:** 10.1038/bjc.1949.36

**Published:** 1949-06

**Authors:** G. Calcutt

## Abstract

**Images:**


### 3:4-BENZPYRENE AS AN –SH INHIBITOR.

G. CALCUTT.

*From the Department of Cancer Research, Mount Vernon Hospital, and the Radium Institute, Northwood, Middlesex.*

Received for publication April 16, 1949

THE question of the involvement of the polycyclic hydrocarbons with sulphydryl groups during the process of carcinogenesis has been under discussion for a long time. Certain indirect evidence exists and has been reviewed by Crabtree (1947), who concludes that the available evidence does not warrant any decision as to whether the involvement is direct or not.

In other directions Mueller and Rusch (1943) could obtain no effect upon the activity of urease—this being dependent upon the integrity of the –SH groups—with benzpyrene solution. On the other hand Rondoni and Bassi (1948) have found benzpyrene to inhibit the papainaise digestion of gelatin and also the cathepsins of rat sarcoma and horse liver. The non-carcinogenic hydrocarbons, phenanthrene, anthracene and pyrene, had no action. Calcutt and Newhouse (1948) also found that the photodynamic action of benzpyrene was inhibited by cysteine hydrochloride.

Further work (Calcutt, 1948) showed that certain other –SH-containing compounds were also effective as inhibitors of photodynamic action. In the attempt to find the mechanism of this action it was found that after incubation of a colloidal suspension of benzpyrene in a solution of cysteine hydrochloride the characteristic yellow-green fluorescence colour of the benzpyrene changed to pale blue. Preliminary attempts to isolate this blue fluorescing compound were unsuccessful, but it was found that the mixture no longer gave a positive test with sodium nitroprusside. This implied the inhibition of the –SH group of the cysteine and formed the starting-point for further work.

### *The inhibition of –SH groups.*

Using colloidal benzpyrene in distilled water, tests were carried out with a series of compounds. In each case the technique was the same. Equal amounts of a solution of the sulphydryl-containing compound were placed in each of two tubes. To one was added some benzpyrene colloid, and to the other an equivalent amount of distilled water. Both were then incubated, and samples from each were tested at intervals with nitroprusside. In this way it was found that inhibition of the –SH of cysteine hydrochloride, thiomalic acid, thioglycollic acid and 2-mercaptoethanol occurred in the tubes containing benzpyrene. In each case the control tube continued to give a positive reaction with the nitroprusside. The inhibition of the –SH appeared to run parallel with a change in fluorescence colour of the benzpyrene from yellow-green to pale blue. There was no apparent fluorescence in the water which acted as the medium.

In the above instances the reaction mixture was in each case acid. So the investigation was extended to cover a greater pH range. Using cysteine, sodium thiomalate, sodium thioglycollate solutions to which varying amounts of sodium hydroxide solutions had been added, it was found that inhibition occurred as before. With slightly alkaline mixtures, however, a small amount of blue fluorescing compound was obtained in solution.

For all the cases mentioned above incubation has to be continued for upwards of $1\frac{1}{2}$ hours at a temperature of 65° C. before any inhibition became apparent. At lower temperatures the action was very slow, and at room temperature—25° C. —there appeared to be no effect whatever.

Preliminary trials having indicated that caffeine solutions had little effect upon –SH as detected by nitroprusside, some of the previous experiments were repeated, using a solution of benzpyrene in a 3 per cent caffeine solution. Inhibition of –SH was achieved as before, but this time much more rapidly—30 minutes at 65° C.

*Isolation of the reaction products.*

Under the conditions so far investigated the yield of the pale blue fluorescing product of the reactions has only been very small. Nevertheless it has been found possible to isolate products from both the alkaline and acid reaction mixtures. The methods used and found suitable were those developed by Weigert and Mottram (1946) and Weigert (1948) for the separation of benzpyrene metabolites as found in animals. In this way small yields of solutions of apparently pure substances were obtained, the final purifications being by repeated chromatography.

*Properties of the reaction products.*

To date insufficient material has been available for chemical analysis, but certain general properties are apparent. From the alkaline reaction mixture a water soluble compound was derived. This is soluble in organic reagents, strongly adsorbed to silica, strongly adsorbed to alumina, fluoresces a whitish-blue colour in ultra-violet light. On addition of dilute acid it changes to a blue fluorescing compound which is soluble in organic reagents, insoluble in water but soluble in dilute alkali.

From the acid reaction mixture a compound has been separated which is soluble in organic reagents, soluble in dilute alkali, insoluble in water, adsorbed strongly to alumina and is weakly adsorbed to silica, moving down slowly as elution is continued.

Thus in their physical properties these compounds show a remarkable similarity to those separated from animals and designated by Weigert and Mottram (1946) as $BpX_1$, $BpF_1$ and $BpF_2$, this latter apparently being identical with the compound characterized by Berenblum, Crowfoot, Holiday and Schoental (1943) as 8.OH Benzpyrene.

Examination of the fluorescence spectra of the derived compounds offers further interest. That of the water soluble compound is found to be comparable to that of $BpX_1$, whilst the alkali soluble product derived by acidification gives a spectrum similar to that of $BpF_1$. In this connection it must be recalled that Weigert and Mottram (1946) found that acid hydrolysis of $BpX_1$ gave rise to $BpF_1$.

The spectrum of the alkali soluble product from the acid reaction mixture resembles that of $BpF_2$ (8.OH.Bp). Furthermore it is found that continued

Fig. 1.—Fluorescence spectra of benzpyrene derivatives: (1) Benzpyrene $X_1$ from mouse. (2) Water soluble compound from alkaline reaction mixture. (3) Benzpyrene $F_1$ from mouse. (4) Alkali soluble product from acidification of (2). (5) 8.OH benzpyrene from mouse. (6) Alkali soluble product from acid reaction mixture.

acid hydrolysis of the compound derived from the water soluble product gives rise to a further compound, which from its general behaviour and fluorescence spectrum appears to be identical with the product derived from the acid reaction.

For purposes of comparison the fluorescence spectra of the compounds obtained as above are shown together with those of $BpX_1$, $BpF_1$ and $BpF_2$ in Fig. 1.

### DISCUSSION.

The work described above indicates that 3 : 4-benzpyrene can engage in direct chemical action with a variety of compounds containing sulphydryl. Furthermore the evidence suggests a considerable parallelism, even if not identity, with the course of events after the introduction of benzpyrene into the animal body. Whilst the apparent identity of the fluorescence spectra does not imply strict chemical identity, it does mean the very close relationship of the substances concerned.

From this it is reasonable to suggest that the interaction of benzpyrene with a –SH containing compound gives rise to a substance of the $BpX_1$ type. This, however, is in the presence of acid successively broken to compounds of $BpF_1$ and $BpF_2$ type. It has already been suggested by Crabtree (1947) that the detoxication of benzpyrene is by way of –SH compounds. In view of the evidence presented above this must now be regarded as being most probable.

Crabtree (1940, 1944, 1945) has as a result of his work with –SH inhibitors suggested the involvement of sulphur in carcinogenesis caused by the polycyclic hydrocarbons. This is further reinforced by the recent work of Lusky, Braun and Woodard (1947), who inhibited the carcinogenic action of the benzpyrene by application of B.A.L. (2:3-dimercaptopropanol). Additionally, evidence has been put forward by Weigert, Calcutt and Powell (1947) to the effect that detoxication occurs at the site of carcinogenesis. Now in view of the above evidence it appears most likely that detoxication and carcinogenesis both occur together, and that both processes are intimately concerned with sulphydryl.

Further evidence is required to reinforce the views expressed above, and to this end an investigation of a series of carcinogenic and non-carcinogenic hydrocarbons has been started whilst work on the chemistry of the reactions described is continuing. So far the data available indicates that other carcinogenic hydrocarbons react with –SH containing compounds in a fashion similar to benzpyrene, but that the non-carcinogenic ones show no similar activity. Full details will be published as soon as possible.

It is futile to speculate further on the role of sulphydryl in carcinogenesis, but in conclusion it is perhaps interesting to recall that Potter (1943) has postulated carcinogenesis as arising by production of abnormal protein ; Rondoni (1948) suggests the action of carcinogenic agents on nucleo- or liponucleo-protein, whilst Powell (1946, 1947) has put forward a hypothesis involving cytoskeletal protein with particular reference to thiol groups. In any case, sulphydryl is inextricably involved in discussions of proteins.

### SUMMARY.

An account is given of the conditions under which 3:4-benzpyrene is found to inhibit the –SH groups of cysteine hydrochlroide, thiomalic acid, thioglycollic acid and 2-mercaptoethanol. The isolation and further examination of reaction products is described. On the basis of physical properties and fluorescence spectra these products are shown to be similar to the benzpyrene metabolites obtained from animals. The relationship of the above findings to carcinogenesis and detoxication are discussed.

The expenses of this research were defrayed out of a block grant from the British Empire Cancer Campaign.

### REFERENCES.

BERENBLUM, I., CROWFOOT, D., HOLIDAY, E. R., AND SCHOENTAL, R.—(1943) *Cancer Res.*, **3**, 151.

CALCUTT, G.—(1948) In press.

*Idem* AND NEWHOUSE, J. P.—(1948) *Nature*, **161**, 53.

CRABTREE, H. G.—(1940) *J. Path. Bact.*, **51**, 303.—(1944) *Cancer Res.*, **4**, 688.—(1945) *Ibid.*, **5**, 346.—(1947) *Brit. med. Bull.*, **4**, 345.

LUSKY, L. M., BRAUN, H. A., AND WOODARD, G.—(1947) *Cancer Res.*, **7**, 667.

MUELLER, G. C., AND RUSCH, H. P.—(1943) *Ibid.*, **3**, 113.

POTTER, V. R.—(1943) *Ibid.*, **3**, 358.

POWELL, A. K.—(1946) *J. Roy. Micro. Soc.*, **66**, 35.—(1947) *Ibid.*, **67**, 14.

RONDONI, P.—(1948) *Schweiz. med. Wschr.*, **78**, 419.

*Idem* AND BASSI, M.—(1948) *Ric. Sci.*, **18**, 8.

WEIGERT, F.—(1948) *Cancer Res.*, **8**, 169.

*Idem*, CALCUTT, G., AND POWELL, A. K.—(1947) *Brit. J. Cancer*, **1**, 405.

*Idem* AND MOTTRAM, J. C.—(1946) *Cancer Res.*, **6**, 97.